# Phosphoproteomic Characterization and Kinase Signature Predict Response to Venetoclax Plus 3+7 Chemotherapy in Acute Myeloid Leukemia

**DOI:** 10.1002/advs.202305885

**Published:** 2023-12-31

**Authors:** Jie Jin, Shangyu Hou, Yiyi Yao, Miaomiao Liu, Liping Mao, Min Yang, Hongyan Tong, Tao Zeng, Jinyan Huang, Yinghui Zhu, Huafeng Wang

**Affiliations:** ^1^ Department of Hematology the First Affiliated Hospital Zhejiang University School of Medicine Hangzhou 310003 P. R. China; ^2^ Zhejiang Provincial Key Lab of Hematopoietic Malignancy Zhejiang University Hangzhou Zhejiang P. R. China; ^3^ Zhejiang Provincial Clinical Research Center for Hematological Disorders Hangzhou China; ^4^ Zhejiang University Cancer Center Hangzhou Zhejiang P. R. China; ^5^ Jinan Microecological Biomedicine Shandong Laboratory Jinan P. R. China; ^6^ Research Center for Translational Medicine Shanghai East Hospital School of Life Sciences and Technology Tongji University Shanghai 200092 P.R. China; ^7^ Biomedical big data center the First Affiliated Hospital Zhejiang University School of Medicine Hangzhou, Zhejiang 310003 P.R. China; ^8^ Frontier Science Center for Stem Cell Research Shanghai Key Laboratory of Signaling and Disease Research Tongji University Shanghai 200092 P.R. China

**Keywords:** acute myeloid leukemia, AKT1, AURKB, drug resistance, venetoclax

## Abstract

Resistance to chemotherapy remains a formidable obstacle in acute myeloid leukemia (AML) therapeutic management, necessitating the exploration of optimal strategies to maximize therapeutic benefits. Venetoclax with 3+7 daunorubicin and cytarabine (DAV regimen) in young adult de novo AML patients is evaluated. 90% of treated patients achieved complete remission, underscoring the potential of this regimen as a compelling therapeutic intervention. To elucidate underlying mechanisms governing response to DAV in AML, quantitative phosphoproteomics to discern distinct molecular signatures characterizing a subset of DAV‐sensitive patients is used. Cluster analysis reveals an enrichment of phosphoproteins implicated in chromatin organization and RNA processing within DAV‐susceptible and DA‐resistant AML patients. Furthermore, kinase activity profiling identifies AURKB as a candidate indicator of DAV regimen efficacy in DA‐resistant AML due to AURKB activation. Intriguingly, AML cells overexpressing AURKB exhibit attenuated MCL*‐1* expression, rendering them receptive to DAV treatment and maintaining them resistant to DA treatment. Moreover, the dataset delineates a shared kinase, AKT1, associated with DAV response. Notably, AKT1 inhibition augments the antileukemic efficacy of DAV treatment in AML. Overall, this phosphoproteomic study identifies the role of AURKB as a predictive biomarker for DA, but not DAV, resistance and proposes a promising strategy to counteract therapy resistance in AML.

## Graphical abstract

The DAV (DA plus venetoclax) regimen as a novel potent therapy, yields a remarkable 90% complete remission rate in adult AML patients. Quantitative phosphoproteomics unveils AURKB as a novel molecular indicator for DA resistance, with DAV restoring sensitivity. Targeting AKT1 emerges as a potential therapeutic strategy to overcome DAV resistance in newly diagnosed AML patients.

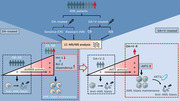

## 1 Introduction

Acute myeloid leukemia (AML) is a heterogeneous hematopoietic disorder characterized by uncontrolled expansion of myeloid stem/progenitor cells, which is accompanied by differentiation blockade.^[^ ^1^ ^]^ Overcoming the challenge of refractoriness after induction chemotherapy and effectively managing AML relapse following remission remain formidable clinical obstacles.^[^ ^2^, ^3^ ^]^ The established daunorubicin and cytarabine (DA) regimen, a mainstay of AML induction therapy for several decades, has demonstrated complete remission rates ranging from ≈60% to 80%, concomitant with a 5‐year overall survival rate of <50%.^[^ ^4^ ^]^ Concomitantly, novel therapeutic avenues have emerged, characterized by a focus on ameliorating the effects of dysregulated and activated proteins that are central to AML pathogenesis. Some of these interventions include inhibitors targeting IDH1/2,^[^ ^5^, ^6^ ^]^ FLT3,^[^ ^7^, ^8^ ^]^ and BCL2.^[^ ^9^ ^]^

Venetoclax, an orally administered selective small molecule inhibitor targeting BCL2,^[^ ^10^ ^]^ has garnered considerable scientific interest. In a recent phase 2 clinical trial, we embarked on meticulously evaluating the therapeutic efficacy of the DAV regimen (a composite of intravenous daunorubicin and cytarabine in combination with venetoclax) in a cohort of adult patients with AML (n = 33).^[^ ^11^ ^]^ Impressively, after a single cycle of DAV induction therapy, a substantial subset of 30 patients attained complete remission, substantiating the potential of the regimen as a compelling therapeutic avenue for the early diagnosis of adult AML cases. Several genomic biomarkers were associated with venetoclax sensitivity, such as *SRSF2* and *IDH* mutations,^[^ ^12^ ^]^ which may predict the sensitivity to venetoclax therapy in AML. Understanding the indication characteristics of the DAV compared to the DA regimen could facilitate the development of optimal chemotherapeutic strategies for AML. Nevertheless, within the scope of our clinical investigation, 3 out of 33 patients were unresponsive to the initial cycle of the DAV regimen. These three patients were characterized by the presence of adverse‐risk genetic mutations in *TP53* and *FLT3‐TKD*, raising the need to investigate the underlying mechanisms of DAV resistance and identify potential methods to overcome it.

The genome and transcriptome have been comprehensively explored to elucidate the intricacies of AML biology and clinical heterogeneity.^[^ ^12^, ^13^, ^14^ ^]^ In contrast, studies focusing on the proteome and phosphoproteome have been relatively limited, albeit with promising insights.^[^ ^15^, ^16^ ^]^ Particularly notable is the distinct vantage point afforded by the phosphoproteome, which provides an encompassing and distinctive outlook on activated signaling cascades that are not readily observable within the transcriptome landscape.

To elucidate the mechanisms governing the therapeutic action and intrinsic resistance in AML following DAV treatment, we performed phosphoproteomic profiling on 10 patients with AML who achieved complete remission (CR) after one cycle of the DAV regimen and six patients who showed no response (NR). Using high‐accuracy mass spectrometry techniques, we established another cohort consisting of ten patients with CR and seven patients with NR, treated with the conventional DA regimen to determine the applicable population for the DAV regimen. Our analysis revealed that phosphoproteins involved in chromatin organization and RNA processing were significantly enriched both in patients with DA‐NR and in those with DAV‐CR. Furthermore, comprehensive kinase analysis highlighted AURKB as a pivotal kinase that orchestrates mitosis, which causes DA resistance, suggesting that AURKB could serve as a potential sensor for DA regimen in de novo AML patients, as well as DAV regimen in AML patients exhibited resistance to DA regimen caused by AURKB activation. Moreover, an in‐depth exploration of the differential phosphoproteomic signatures of patients with DAV‐CR or DAV‐NR revealed distinct enrichment of energy metabolism‐associated kinases, including OSXR1, CK2, and AKT1, within the DAV‐resistant patient subsets. To address DAV resistance, we extended our investigation to evaluate the efficacy of an AKT1 inhibitor, which significantly diminished resistance across AML lines and primary cells in vitro. Collectively, these multifaceted findings provide a novel molecular indication for the DAV regimen in newly diagnosed patients with AML while concurrently proposing a potential therapeutic strategy to overcome DAV resistance and enhance clinical efficacy.

## 2 Experimental Section

### 2.1 Patient and Sample Collection

The characteristics of all the patients are summarized in **Table** **1** and Table S1 (Supporting Information). To obtain samples for analysis, mononuclear cells were isolated from each patient's bone marrow (BM) at the time of diagnosis. Sample acquisition was conducted following the guidelines of the Declaration of Helsinki and approved by the Institutional Review Board of the First Affiliated Hospital of Zhejiang University School of Medicine. Prior to inclusion in the study, written informed consent was obtained from all participants to ensure their understanding and willingness to participate.

**Table 1:** Statistical consideration of variation among complete remission (CR) and non‐response (NR) patients from DA or DAV group.

Characteristics	DA‐CR (*N* = 10)	DA‐NR (*N* = 7)	DAV‐CR (*N* = 10)	DAV‐NR (*N* = 6)	P_1_/P_2_
Age (years, range)	33.4 (21–42)	44 (28–58)	34.4 (19–49)	50.5 (32–64)	0.034/0.017
Gender (M/F)	5/5	2/5	5/5	5/1	0.622/0.307
WBC (10^9^/L, range)	46.58 (0.5–153.5)	49.50 (0.61–141.9)	70.3 (4.2–209.85)	37.19 (11.07–67.75)	0.908/0.229
N (10^9^/L, range)	5.83 (0.08–21)	4.58 (0.09–10.31)	14.5 (0.19–95.03)	9.83 (0.27–23.81)	0.887/0.792
HGB (g/L, range)	85.1 (58–134)	84.43 (64–116)	100.7 (73–123)	86.67 (61–145)	0.951/0.181
PLT (10^9^/L, range)	41.1 (13–95)	42.71 (11–99)	61.7 (13–187)	60.83 (19–115)	0.740/0.958
BM blasts (%, range)	75.5 (50–92)	74.21 (63.5–82)	78.8 (63–92)	59.67 (27–89)	0.475/0.29
Karyotype risk stratification					0.403/0.332
Favorable (%)	20	28.57	10	0	
Intermediate (%)	80	57.14	60	100	
Adverse (%)	0	14.29	30	0	
ELN risk stratification					1.000/0.091
Favorable (%)	50	42.86	50	0	
Intermediate (%)	30	42.86	10	50	
Adverse (%)	20	14.29	40	50	

DA, Darubicin+Cytarabine; DAV: Darubicin+Cytarabine+Venetoclax; F, female; M, male; WBC, white blood cell count; N, neutrophil count; HGB, hemoglobin; PLT, platelet; BM, bone marrow; FCM, flow cytometry; ELN, European Leukemia Network; P1, companion between DA‐CR and DA‐NR; P2:companion between DAV‐CR and DAV‐NR.

### 2.2 Sample Preparation for Mass Spectrometry Analysis

#### 2.2.1 Protein Extraction and Digestion

For protein extraction, lysis buffer containing 8 m urea, 1% protease inhibitor cocktail, and 1% phosphatase inhibitor was added to the samples, and these were sonicated five times (5 s “on” and 5 s “off”) on ice using a high‐intensity ultrasonic processor. The remaining debris was removed by centrifugation at 12000×g at 4 °C for 10 min. Finally, the supernatant was harvested, and the protein concentration was determined with a BCA kit. For protein digestion, the protein solution was reduced by the addition of 5 mm dithiothreitol and incubation at 56 °C for 30 min. Next, 11 mm iodoacetamide was added to alkylate the proteins, and the mixtures were incubated for 15 min at room temperature in the dark. The protein sample was then diluted by adding 100 mm tetraethylammonium bromide (TEAB) to reduce the urea concentration to less than 2 m. Finally, trypsin was added at a 1:50 trypsin‐to‐protein mass ratio for the first digestion overnight and a 1:100 trypsin‐to‐protein mass ratio for a second 4 h digestion. Finally, the peptides were desalted using a C18 SPE column for tandem mass tag (TMT) labeling.

#### 2.2.2 TMT 10‐Plex Labeling

The isobaric labeling experiment was conducted according to the TMT kit instructions. For each TMT 10‐plex labeling experiment, each channel was labeled with 550 µg of peptides. All 33 samples were divided into four groups; 8–9 samples were labeled with channels 126, 127N, 127C, 128N, 128C, 129N, 130N, and 130C. For the “internal reference,” mixed samples were used in TMT labeling, and 50 µg of samples from each group were labeled with channel 131N. Tryptic peptides were first dissolved in 0.5 m TEAB. Each peptide channel was labeled with its respective TMT reagent (Thermo Fisher Scientific, Rockfolr, IL, USA) based on the manufacturer's protocol and incubated for 2 h at room temperature. 5 µL of each sample were pooled, desalted, and analyzed by mass spectrometry (MS) to determine the labeling efficiency, after which they were quenched by adding 5% hydroxylamine. Pooled samples were desalted using a Strata X C18 SPE column (Phenomenex, Torrance, CA, USA) and dried by vacuum centrifugation.

#### 2.2.3 High‐pH High‐Performance Liquid Chromatography Fractionation

The sample was fractionated by high‐pH reverse‐phase high‐performance liquid chromatography using an Agilent 300 Extend C18 column (5 µm particles, 4.6 mm ID, 250 mm length). Briefly, peptides were separated using a gradient of 2–60% acetonitrile in 10 m ammonium bicarbonate (pH 10) for 80 min into 80 fractions. The peptides were combined into nine fractions and dried by vacuum centrifugation.

#### 2.2.4 Phosphopeptide Enrichment

Peptide mixtures were first incubated with an IMAC microsphere suspension under vibration in a loading buffer (50% acetonitrile/0.5% acetic acid). To remove nonspecifically adsorbed peptides, the IMAC microspheres were washed with 50% acetonitrile/0.5% acetic acid and 30% acetonitrile/0.1% trifluoroacetic acid. To elute the enriched phosphopeptides, an elution buffer containing 10% NH_4_OH was added and the enriched phosphopeptides were eluted under vibration. The supernatant containing the phosphopeptides was collected and lyophilized for liquid chromatography (LC)‐MS/MS analysis.

### 2.3 LC‐MS/MS Analysis

For phosphoproteomic analysis, the tryptic peptides were dissolved in solvent A (0.1% formic acid, 2% acetonitrile in water) and directly loaded onto a home‐made reversed‐phase analytical column (25 cm length, 75 µm i.d.). Peptides were separated with a gradient of 5–25% solvent B (0.1% formic acid in 90% acetonitrile) over 60 min, 25–35% over 22 min, 80% over 4 min, and then held at 80% for the last 4 min, all at a constant flow rate of 450 nL min^−1^ using an EASY‐nLC 1200 UPLC system. The separated peptides were analyzed using an Orbitrap Exploris 480 (Thermo Fisher Scientific) with a nano‐electrospray ion source. The applied electrospray voltage was 2.1 kV. The full MS scan resolution was set to 60 000 for a scan range of 350–1400 m/z. Up to 20 of the most abundant precursors were selected for further MS/MS analysis with 30 s of dynamic exclusion. High‐energy collisional dissociation fragmentation was performed at a normalized collision energy of 28%. Fragments were detected using Orbitrap at a resolution of 30 000. The first fixed mass was set at 110 m/z. The automatic gain control (AGC) target was set at 1E5, with an intensity threshold of 1E4 and a maximum injection time of 50 ms.

### 2.4 Database Searching

The resulting MS/MS data were processed using the MaxQuant search engine (v.1.6.15.0). Tandem mass spectra were searched against the human Swiss‐Prot database and concatenated with a reverse‐decoy database. Trypsin/P was used as the cleavage enzyme, allowing for up to two missing cleavages. The mass tolerance for the precursor ions was set to 20 ppm in the first search and 5 ppm in the main search. The mass tolerance for the fragment ions was set to 0.02 Da. Carbamidomethylation on Cys was specified as a fixed modification, and acetylation at the protein N‐terminus and oxidation on Met were specified as variable modifications. The false discovery rate was adjusted to <1%.

### 2.5 Western Blotting

Cells were lysed in a buffer containing 50 mm Tris (pH 7.4), 150 mm NaCl, 1 mM EDTA, 0.5% NP40, and 0.5% sodium deoxycholate supplemented with protease and phosphatase inhibitors. Boiled lysates were resolved on 7.5% sodium dodecyl sulfate‐polyacrylamide gel electrophoresis (SDS‐PAGE) gels and transferred to nitrocellulose membranes, prior to incubation with primary and secondary antibodies. Horseradish peroxidase‐conjugated secondary antibodies were purchased from Jackson ImmunoResearch Laboratories (West Grove, PA, USA). Antibody detection was performed using the SuperSignal West Pico or Femato kits (Thermo Fisher Scientific, Rockfolr, IL, USA), and the results were imaged by G:BOX Chemi XX6 gel doc systems (Syngene) and visualized using GeneSys image acquisition software (Syngene). Protein levels were determined by densitometry using ImageJ (NIH, Bethesda, MD, USA).

### 2.6 Analysis of Cell Viability and Apoptosis

Cell viability was measured using the Cell Titer‐Glo Luminescent Cell Viability Assay kit (Promega, Madison, WI, USA) in accordance with the manufacturer's instructions. Each experiment was performed in triplicate. For apoptosis, cells were labeled with Annexin V/DAPI and analyzed by flow cytometry (LSRII; BD).

### 2.7 RNA‐Seq Analysis

Sequencing libraries were constructed using the VAHTS Universal V8 RNA‐seq Library Prep Kit for Illumina (Vazyme Biotech, NR605‐02) according to the manufacturer's instructions. All libraries were sequenced on an Illumina NovaSeq 6000 platform with a 150‐bp paired‐end module. Data preprocessing followed the GATK best practices. The original gene expression matrix and corresponding fragment per kilobase per million (FPKM) expression matrix were generated using Salmon (v1.9.0). Differential expression analysis within the cell lines was performed using DESeq2 (v1.40.2). Differentially expressed genes (p adj<0.05, and abs (log FC)>1) were selected for gene ontology (GO)/Kyoto Encyclopedia of Genes and Genomes enrichment analysis. Finally, the FPKM expression matrix was used to remove low‐expression genes and Gene Set Enrichment Analysis (GSEA) (v4.3.2) analysis was performed.

### 2.8 Data Sharing Statement

The data reported in this paper were deposited to OMIX, China National Center for Bioinformation/Beijing Institute of Genomics, Chinese Academy of Sciences (https://ngdc.cncb.ac.cn/omix: accession no. OMIX 004738).

### 2.9 Statistical Analysis

Statistical analyses were performed as indicated in each figure using GraphPad Prism software (v9). A one‐way ANOVA with multiple comparisons was used to compare multiple groups of cell lines and primary patient groups. A *p* value of <0.05 was considered statistically significant(*, *p*<0.05; **, *p*<0.01; ***, *p*<0.001, and ****, *p*<0.0001), and data are presented as mean ± SEM.

## 3 Results

### 3.1 Quantitative Phosphoproteomics Profiling Reveals Conserved Signature Linked to DAV Regimen Response in AML

**Figure 1:**
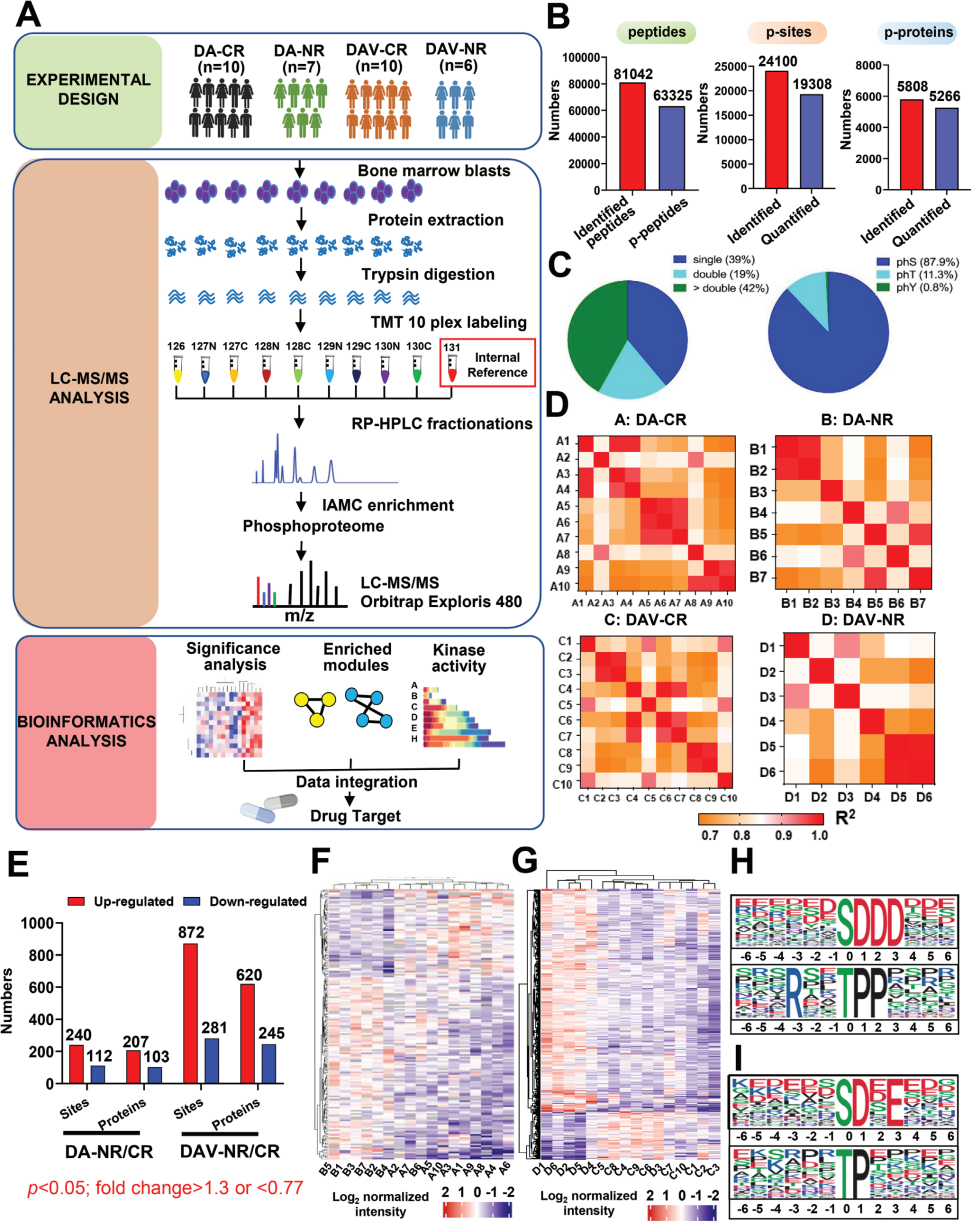
Quantitative phosphoproteomics profiling reveals a conserved signature linked to DAV regimen response in AML. A) Experimental design and phosphoproteomics workflow used to investigate the sensitivity and resistance to DAV in AML. Patients were categorized into complete remission (CR) and non‐response (NR). B) Number of phosphoproteins, phospho‐sites, and phosphopeptides identified and quantified in this study. C) Distribution of phosphopeptides with one, two, or more phosphorylated sites. Distribution of phosphorylated serine (S), threonine (T), and tyrosine (Y) sites in bone marrow cells from 33 patients with AML. D) Heatmap showing Pearson's correlation coefficients (R2) of phospho‐proteome data, indicating reproducibility among individual BM cells from patients with AML among CR or NR groups. E) Summary of differential phosphoproteins identified in comparisons of DA‐NR versus CR and DAV‐NR versus CR groups. The criteria for differential phosphoproteins were a fold change (NR/CR) of >1.3 or <0.67 and *p*<0.05. The selection of differential proteins was made from a pool of 3804 phosphoproteins that were both identified and quantified in all four patient groups. F, G) Heatmaps showing the normalized abundance of differential phosphoproteins through unsupervised hierarchical clustering in 33 individual samples. Most specimens exhibited distinct unsupervised hierarchical clustering in 33 individual samples. H, I) Motif analysis of the phospho‐sites, with top phosphorylation motif of S, T enriched in total identified phosphorylated proteins (H), and differential phosphoproteins in DAV‐NR/CR (I). The numbers displayed under each motif indicate the positions upstream and downstream relative to the central phosphorylation site.

To gain insights into the molecular mechanisms associated with the efficacy of the DAV regimen in patients newly diagnosed with AML, we adopted a quantitative phosphoproteomics methodology. Bone marrow specimens were collected from 33 newly diagnosed patients with AML with comparable clinical characteristics (Tables 1; Table S1, Supporting Information), who had undergone either DA or DAV induction therapy. The patients were divided into four categories according to whether they demonstrated complete remission (CR) or no response (NR) after DA or DAV induction therapy: DA‐CR (*n* = 10), DA‐NR (*n* = 7), DAV‐CR (*n* = 10), and DAV‐NR (*n* = 6). Using isobaric TMT‐based global phosphoproteomics (**Figure** **1**A), we identified 24 100 highly reliable phospho‐sites in 5808 phosphoproteins and 63325 phosphopeptides (Figure 1B; Table S2, Supporting Information). MS data were of high quality, as indicated by peptide lengths of 7–20 aa and a phosphopeptide distribution of 300–1200 m/z (Figures 1C; Figure S1A, Supporting Information). Linear regression analysis was performed among the individual cohorts, and the correlation heatmap displayed good reproducibility for phosphopeptides (Figure 1D).

Our analysis identified 310 regulated phosphoproteins, 207 of which were significantly more abundant in the DA‐NR group, and 103 were upregulated in the DA‐CR group. Strikingly, 865 phosphoproteins were upregulated, 620 of which were prominently upregulated in DAV‐resistant patients (DAV‐NR) (Figure 1E; Table S3, Supporting Information). These findings strongly suggested there is an orchestrated mechanism of resistance to the DAV regimen in AML compared to the DA regimen. Unsupervised hierarchical clustering analysis of differential phosphoproteins separated resistant (NR) from sensitive (CR) patients in both DA (Figure 1F) and DAV groups (Figure 1G). This distinct phosphoprotein signature in patients with chemo‐failure indicates the presence of potential molecular markers associated with treatment response. Patient B6 from the DA‐NR group and patient D3 from the DAV‐NR group were isolated from their respective clusters, which could be attributed to the high white blood cell count or other disease‐related factors prevalent in these patients.

To further explore the potential kinase‐relevant motifs associated with DAV resistance in AML, we examined the enriched phosphorylated motifs in the identified phosphopeptides. We observed that xxSDDDxx and xxSPxKxxK phosphorylation motifs were enriched in all identified phosphoproteins (Figure 1H; Figure S1B,C, Supporting Information), whereas xxxxxx_S_DxExxx and xxxxxx_T_Pxxxxx phosphorylated motifs were enriched in the regulated proteins associated with DAV resistance (Figure 1I; Figure S1D,E, Supporting Information).

### 3.2 Kinase Signature Associated with Treatment Response to DA and DAV Regimen Pinpoints AURKB Activation

**Figure 2:**
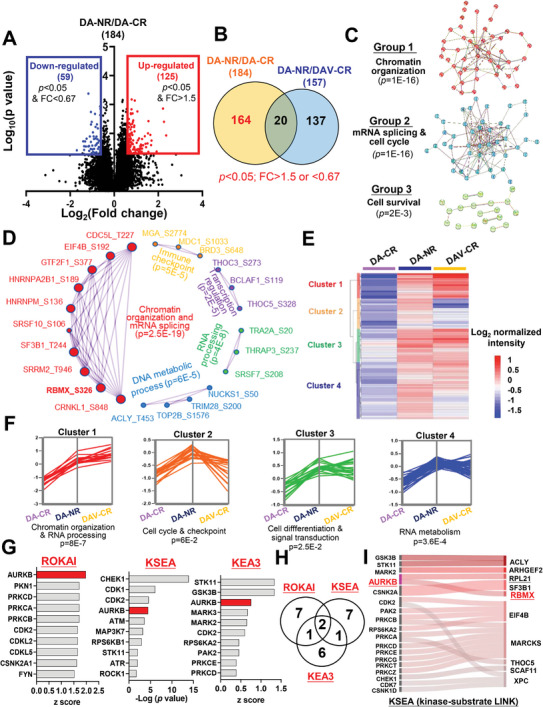
Kinase signature associated with treatment response to DA and DAV regimen pinpoint AURKB activation. A) Volcano plot illustrating the distribution of phosphorylated proteins with their relative protein abundance in the DA‐NR and DA‐CR groups. Phosphoproteins with a fold change (DA‐NR/CR) of >1.5 or <0.67 and a significance level (*p‐value*) of <0.05 are depicted in red (increased) or blue (decreased). B) Venn diagram comparing the differential phosphoprotein datasets between the DA‐NR/DA‐CR and DA‐NR/DAV‐CR groups. C) Functional protein‐protein interaction (PPI) network of enriched phosphoproteins associated with DAV regimen susceptibility. The network was generated using stringAPP and clusters using MCL clustering. An analysis of Gene Ontology enrichment was conducted, yielding four distinct groupings. D) PPI network and significant models constructed by Metascape analysis. E) Hierarchical clustering of 164 significantly regulated proteins potentially involved in DAV sensitivity according to their respective expression level across the DA‐CR, DA‐NR, and DAV‐CR groups. F) Protein expression profiles for each cluster were shown on the left, with the most enriched gene ontology term shown at the bottom of each profile. G) Significantly activated kinases associated with the susceptibility of DAV regimens based on ROKAI, KSE1A, and KEA3 analyses. Gary bars represent the enriched analysis, whereas blue bars indicate high confidence and consistent kinases, highlighted by three analysis tools. H) Venn diagram showing the top 10 significant kinases enriched by ROKIA, KSEA, or KEA3 analyses, respectively. I) Sankey diagram displaying the top 10 enriched phosphoproteins (substrates) of the DAV‐sensitivity signature and their corresponding kinases, as determined by KSEA analysis (kinase‐substrate link).

To understand the phospho‐signaling underlying the high clinical efficacy of DAV chemotherapy relative to the DA regimen in AML, we initiated our inquiry by defining the phospho‐signature associated with DA resistance. We identified phosphoproteins that were either upregulated or downregulated in the DA‐NR group relative to those in the DA‐CR using specific criteria (fold change>1.5 or <0.67 and *p*<0.05) (**Figure** **2**A). Building on this foundation, we eliminated differential proteins (DA‐NR vs DAV‐CR) from the DA‐resistance‐characterized phosphoproteins (Figure 2B). The remaining 164 phosphoproteins showed comparative or non‐significant differential expression between the DA‐NR and DAV‐CR groups, suggesting that the patients’ phosphoproteomic patterns displayed resistance to DA but sensitivity to DAV treatment. These phosphoproteins collectively formed a distinctive phospho‐signaling characteristic of the DAV‐applicable population compared to the DA regimen.

Next, we constructed protein–protein association networks using STRING analysis, subsequently clustering the 164 phosphoproteins into three groups (Figure 2C). GO enrichment analysis on each group demonstrated that the most enriched group‐1 proteins were associated with the biological process of “chromatin organization,” whereas group‐2 and group‐3 exhibited enrichment in phosphoproteins corresponding to mRNA splicing and the cell cycle or cell survival, respectively. Further, pathway enrichment analysis using Metascape highlighted the prominence of diverse DNA/RNA metabolic processes, including chromatin organization, mRNA splicing, DNA metabolic processes, and RNA processing (Figure 2D). Notably, phosphoproteins with known regulatory functions in chromatin organization, such as CDC5L, EIF4B, GTF2F, HNRNPA2B1, HNRNPM, SRSF10, SF3B1, SRRM2, RBMX, and CRNKL1 were gradually enriched for DAV sensitivity.

To further elucidate the specific activated signaling, indicative of DAV sensitivity, while considering DA resistance, we performed hierarchical clustering to ascertain the directionality of the regulation (Figure 2E). We identified four expression clusters (C1–C4; Figure 2F), with C1 being the most enriched in chromatin organization and RNA processing. This finding corroborates and reinforces the results of earlier analyses.

To identify the putative upstream kinases governing DAV signature profiling, we used three distinct kinase‐substrate analysis tools (ROKAI, KSEA, and KEA3), culminating in the identification of the top ten enriched kinases responsible for regulating DAV sensitivity (Figure 2G). Among these candidates, CDK2 and AURKB were identified as the common factors (Figure 2H). CDK2 is a potent cell cycle regulator and a well‐characterized mediator of drug resistance in various cancers, including AML.^[^ ^17^, ^18^ ^]^ Aurora kinase B (AURKB) is a functional serine/threonine kinase involved in chromosome segregation during mitosis and promoting cancer progression.^[^ ^19^, ^20^ ^]^ We further ranked all enriched kinase‐substrate pairs and found that AURKB was responsible for catalyzing RBMX at S326 (Figure 2I), as previously reported.^[^ ^21^, ^22^, ^23^ ^]^ Remarkably, phosphorylated RBMX_S326 expression was notably higher in the DA‐NR and DAV‐CR groups than in the DA‐CR group (log_2_ intensity: 0.004 (DA‐NR), 0.26 (DAV‐CR) vs −0.89 (DA‐NR)). This finding strengthens the hypothesis that AURKB is involved in both DA‐NR and DAV‐CR. Our analysis strongly supports the notion that the DAV regimen shows good clinical efficacy in patients with AML who exhibit resistance to DA caused by elevated AURKB activity.

### 3.3 DAV Regimen Restores the Sensitivity of DA Resistance Caused by AURKB Activation in AML

**Figure 3:**
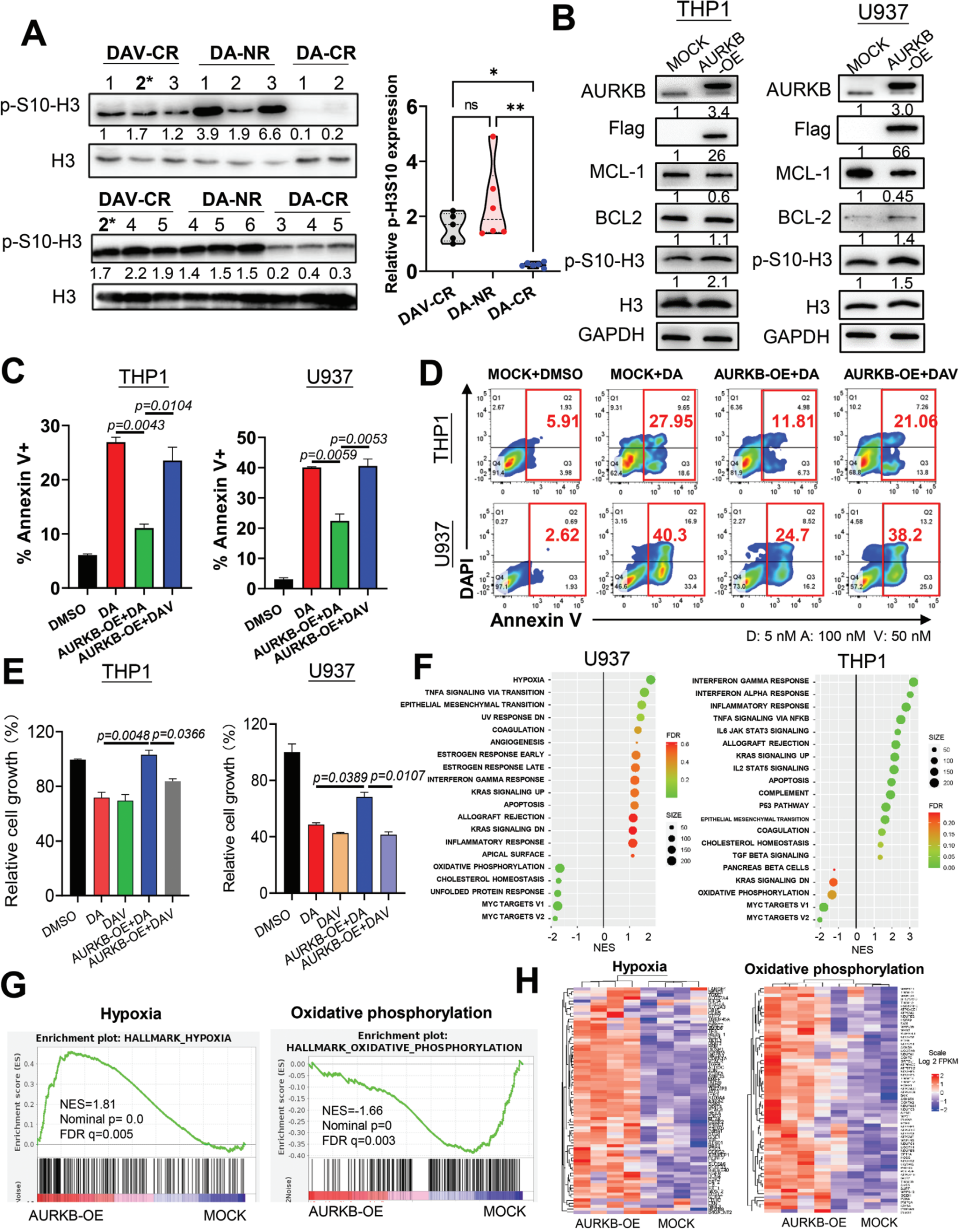
DAV regimen restores the sensitivity of DA resistance caused by AURKB activation in AML. A) Immunoblot analysis of lysates from newly diagnosed AML specimens, showing the levels of phosphorylated Histone 3 at S10 (p‐H3S10) and total Histone 3. DAV‐CR (*n* = 5); DA‐NR (*n* = 6); and DA‐CR (*n* = 5). Protein expression was quantified using the ImageJ software. B) THP1 and U937 cells were transfected with a Flag‐tagged AURKB expression plasmid to ectopically overexpress AURKB. The lysates were immunoblotted for the indicated antibodies. C, D) THP1 or U937 cells with mock or AURKB overexpression were treated with DMSO, DA(D+A), or DAV(D+A+V) [Daunorubicin D): 5 nm, Cytarabine (A): 100 nm, and Venetoclax (V): 50 nM] for 48 h. Cell apoptosis was indicated by Annexin V and DAPI staining (*n* = 3 replicates per sample). E) The cell viability of mock and AURKB‐overexpressing THP‐1 or U937 cells was analyzed after treatment with the indicated drugs for 48 h (*n* = 3 replicates per sample). F) Scattergrams of top hallmark gene sets based on enrichment analyses of expressed genes in U937 (AURKB‐OE versus mock) and THP1 (AURKB‐OE vs mock) The color indicates the false discovery rate q values. NES, normalized enrichment score. G) Gene set enrichment analysis (GSEA) plots of hypoxia that were up‐regulated and oxidative phosphorylation was down‐regulated upon AURKB overexpression in U937 cells. H) The HALLMARK_OXIDATIVE_PHOSPHORYLATION gene set was down‐regulated in the AURKB_OE group of the U937 cell line. The heat map shows the scaled expression of the CORE ENRICHMENT genes of this gene set in both groups. Error bars represent the SEM.

We next focused on Histone 3 S10 (H3S10), a well‐known substrate of AURKB during mitosis,^[^ ^24^, ^25^ ^]^ which serves as an indicator of AURKB activation. We measured the levels of phosphorylated H3S10 in AML specimens of the three groups: DA‐CR (*n* = 5), DA‐NR (*n* = 6), and DAV‐CR (*n* = 5). Intriguingly, we found a substantial elevation in phosphorylated H3S10 both in patients with DA‐NR and in those with DAV‐CR AML compared to that in patients with DA‐CR (**Figure** **3**A).

To further confirm the link between AURKB activation and response to DA and DAV treatment, we knocked down AURKB in primary AML cells that showed relative resistance to DA. The decreased expression of AURKB restored the sensitivity of DA treatment in patients with AML (Figure S2A–C, Supporting Information). Furthermore, we investigated ectopic AURKB expression in THP1 and U937 cells. Notably, AML cells overexpressing AURKB exhibited resistance to DA treatment; however, these cells regained sensitivity when treated with DAV (Figure 3B‐E). Additionally, we observed a decrease in MCL‐1, another important anti‐apoptotic protein, upon AURKB overexpression (Figure 3B). Downregulation of MCL‐1 in AURKB‐overexpressing cells may be responsible for the restoration of sensitivity to DAV treatment. It is possible that AURKB overexpression causes a reduction in MCL*‐1* expression, thereby increasing the reliance on BCL‐2 and consequently overcoming the DA resistance caused by AURKB upon the addition of venetoclax in the DAV regimen.

To further elucidate the potential molecular mechanisms by which the DAV regimen results in cells overcoming the resistance caused by AURKB activation, we overexpressed AURKB in two AML cell lines (U937 and THP1) and analyzed their gene expression by RNA‐seq. GSEA revealed that AURKB overexpression caused the upregulation of gene sets involved in hypoxia and the downregulation of oxidative phosphorylation‐related genes in both cell lines and particularly in U937 cells (Figure 3F**–**H; Figure S2D, Supporting Information). Hypoxia reduced MCL*‐1* expression, which is consistent with MCL‐1 downregulation following AURKB overexpression. Notably, MCL‐1 is known to play a pivotal role in maintaining oxidative phosphorylation, thereby contributing to venetoclax resistance in AML.^[^ ^26^, ^27^ ^]^ Our findings suggest that AURKB induces hypoxic conditions, resulting in decreased MCL‐1 expression. This, in turn, leads to the downregulation of oxidative phosphorylation. Importantly, this alteration in cellular processes may sensitize DA‐resistant cells caused by AURKB activation to DA plus venetoclax (DAV) treatment, potentially overcoming drug resistance. These findings further support that AURKB activation holds potential as a predictive marker for the response of patients with AML to DA and that DAV treatment could overcome the resistance in this context. They also provide an avenue for further research into the mechanistic basis of DA and DAV modulation.

### 3.4 AKT1 Activation is a Specific Marker of AML Resistance to DAV Treatment

**Figure 4:**
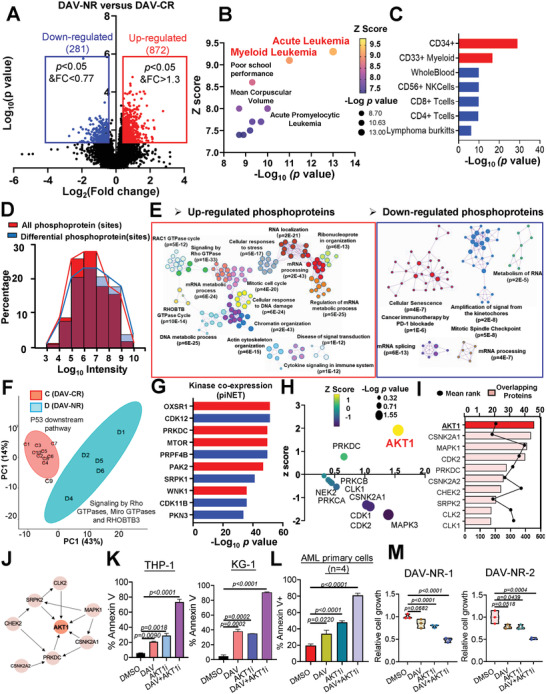
AKT1 activation is a specific marker of AML resistance to DAV treatment. Volcano plot displaying the distribution of phosphorylated proteins with relative protein abundance in DAV‐NR versus DAV‐CR (FC>1.3 or <0.77 and *p*<0.05). B, C) Enrichment analysis of disease (B) and cell type (C) based on differential phosphoproteins in DAV‐NR/CR, according to piNET analysis (a versatile web platform for downstream analysis and visualization of proteomic datasets). D) iBAQ analysis of all identified proteins and differentially expressed phosphoproteins in the DAV‐NR and DAV‐CR groups. E) Functional STRING network of significantly regulated phosphoproteins in patients with DAV‐NR, generated by string APP. Clusters were generated using MCL clusters. F) PCA of differential phosphoproteins between the C (DAV‐CR) and D (DAV‐NR) groups. Different colors indicate different groups (red, DAV‐CR; cyan, DAV‐NR). The ellipse represents an “error ellipse” with a confidence level of 0.95. G) Kinase co‐expression analysis of differentially phosphorylated proteins according to the piNET analysis. H) Predicted top ten enriched kinases based on KSEA analysis of differentially phosphorylated proteins. I, J) Predicted top ten enriched kinases (I) and kinase‐kinase networks (J) based on KEA3 analysis of differentially phosphorylated proteins. K) Apoptosis analysis after 48 h of combination treatment with a‐674563 (AKT1 inhibitor) and/or DAV of THP1 or KG‐1 cells (for both cells: Daunorubicin (D): 5 nm, Cytarabine (A): 100 nm, and Venetoclax (V): 50 nm; a‐674563: THP1 (1 µM) KG‐1 (0.5 µm)) (*n* = 3 replicates per sample). L) Apoptosis analysis after 48 h of combination treatment with a‐674563 (1 µm) and/or DAV (D: 5 nm, A: 100 nm, and V: 50 nm) in AML specimens (*n* = 4). The percentage of apoptotic cells was determined by annexin V staining. M) Cell viability analysis after 48 h of combination treatment with a‐674563 and/or DAV in two AML specimens exhibiting clinical resistance to DAV treatment (*n* = 3 replicates per sample). Error bars represent the SEM.

Although the DAV regimen has demonstrated remarkable clinical efficacy, ≈10% of patients with *de novo* AML have been shown not to respond to DAV treatment.^[^ ^11^ ^]^ Therefore, understanding the underlying mechanisms of DAV resistance and identifying potential methods to improve therapeutic regimens are of utmost importance. We conducted an in‐depth analysis of the phosphoproteome of patients with DAV‐NR and DAV‐CR, which revealed 872 upregulated and 281 downregulated proteins associated with DAV resistance (**Figure** **4**A). Enrichment analysis of all differentially expressed proteins in DisGeNET, a gene‐disease association database, showed that the most enriched diseases were acute leukemia and myeloid leukemia (Figure 4B). Moreover, cell‐type enrichment analysis using piNET (a versatile web platform for downstream analysis)^[^ ^28^ ^]^ indicated that the regulated proteins involved in DAV resistance were specific to CD34^+^ and CD33^+^ myeloid cells (Figure 4C). Interestingly, the differential enrichment in phosphoproteins displayed a higher intensity, as indicated by the right shift in the intensity distribution, indicating their potential significance in DAV resistance (Figure 4D). This suggests that the phosphoprotein signature of DAV resistance is disease‐and cell type‐specific rather than a common characteristic among cancers. These findings provide meaningful directions for overcoming DAV resistance in AML.

Next, we performed functional network analysis, followed by MCL clustering and GO enrichment of significant subclusters among the upregulated proteins associated with DAV resistance (Figure 4E). This revealed enrichment of GO terms such as “Cellular Response to DNA damage,” “Signaling by Rho GTPase,” and “Cytokine signaling in the immune system,” which appeared to be unique to this dataset. To gain further insights into the biological basis of DAV resistance, we conducted a principal component analysis of the phosphoproteome data, and each group showed unique features (Figure 4F). The DAV‐NR group exhibited signaling activation by Rho GTPases, Miro GTPases, and RHOBTB3, whereas the DAV‐CR group showed upregulation of the downstream P53 pathway.

To elucidate the activated kinases involved in DAV resistance, we performed kinase substrate analysis using various powerful tools, including piNET, IPA, KSEA, and KEA3. Five of the top ten kinases were associated with oxidative stress and energy metabolism based on the ARCHS4 co‐expression database (Figure 4G). IPA indicated that the top 10 molecules were activated in patients with DAV‐NR, including kinases CK2, CDK6, SYK, GH1, and CIP2A, which are involved in energy metabolism (Figure S3A, Supporting Information). KSEA analysis suggested that AKT1 was the most enriched upstream kinase, contributing to the differential enrichment in phosphoproteins (Figure 4H). The AKT1 major substrates were consistently upregulated in DAV‐resistant patients (Figure S3B, Supporting Information), and its activity was elevated in a small cohort of DAV‐NR patients, relative to DAV‐CR patients (Figure S3C, Supporting Information). Interestingly, AKT1 also served as the central regulator of all other related kinases and was one of the most activated kinases in the DAV‐NR group (Figure 4I,J). Since AKT1 is hyperactivated in many cancers^[^ ^29^ ^]^ including leukemia, it is a potential signature of DAV resistance. Consequently, AKT1 inhibition by a‐674563 strongly blocked its downstream signaling and significantly enhanced DAV‐induced cell death in AML cells and primary cells from patients with AML (Figure 4K,L; S3D–F, Supporting Information).

To mimic in vivo conditions, we employed primary AML cells that were inherently resistant to DAV treatment. Given the relative rarity of DAV‐resistant patients, we procured samples from two new DAV‐resistant patients and administered a combination of DAV and AKT1 inhibitors. As depicted in Figure 4M and Figure S3G,H (Supporting Information), the AKT1 inhibitor strongly enhanced the anti‐leukemic activity relative to DAV alone in DAV‐resistant primary cells. These findings indicate that AKT1 kinases may play a critical role in DAV resistance and that targeting AKT1 with an inhibitor may be an effective approach to overcome DAV resistance in AML.

## 4 Discussion

Daunorubicin and cytarabine, collectively known as the DA regimen, have been standard induction therapies for adult patients with AML for several decades. However, despite advances in chemotherapy and hematopoietic stem cell transplantation for AML, ≈40% of patients experience refractory or relapsed disease. Therefore, there is an urgent need for effective combined therapeutic regimens.^[^ ^30^, ^31^ ^]^

Venetoclax, an oral BCL‐2 protein inhibitor, has shown promising results in combination with hypomethylating agents or low‐dose cytarabine in older patients with AML.^[^ ^11^, ^32^ ^]^ Various clinical trials have investigated the combination of venetoclax with other targeted therapies for specific AML subgroups, such as *IDH* inhibitors for *IDH1/2*‐mutant AML^[^ ^33^ ^]^ and *FLT3* inhibitors for *FLT3*‐mutant AML.^[^ ^10^, ^34^ ^]^

In a previous study,^[^ ^11^ ^]^ we reported that the DAV regimen was an effective induction therapy for adult patients with AML, resulting in a 91% CR rate in the entire cohort after one cycle. Since then, 72 patients with *de novo* AML received the DAV regimen as induction therapy in our center. Among them, 90.3% (65/72) achieved CR, and 9.7% (7/72) showed NR. Gene mutations in patients with NR included *KRAS, FLT3‐ITD, FLT3‐TKD, TP53, and ASXL1* mutations (Table S1, Supporting Information). In addition, we found that the CR rate after one cycle of DAV treatment was 100% (6/6), 100% (13/13), and 100% (2/2) in patients with AML bearing a *KMT2A* rearrangement, *RUNX1::RUNX1T1*, or *CBFβ::MYH11*, respectively. In patients with AML bearing an *NPM1* mutation, an *IDH1/2* mutation, an *FLT3‐ITD* mutation, an *FLT3‐TKD* mutation, a *NUP98* rearrangement, or a *TP53* mutation, the CR rate after one cycle was 94.4% (17/18), 90.9% (20/22), 89.5% (17/19), 87.5% (7/8), 75.0% (3/4), and 0% (0/1), respectively (Table S4, Supporting Information). Regarding the therapeutic efficacy of DA and DAV treatments in patients with AML and different oncogenic backgrounds, despite the small number of patients in the subgroup, the DAV regimen resulted in a higher CR rate in patients bearing an *NPM1* mutation, a *KMT2A* rearrangement, an *FLT3‐ITD* mutation, or *RUNX1::RUNX1T1* compared to those under the DA regimen. In patients with *NPM1* mutations, the CR rate after one cycle was higher in the DAV group than in the DA group (94.4% vs 54.1%, *p* = 0.018). Moreover, there was a trend toward a higher CR rate after one cycle in the DAV group than in the DA group in patients with AML‐bearing *KMT2A* rearrangements (100% vs 33.3%, *p* = 0.061), *FLT3‐ITD* mutations (89.5% vs 61.5%, *p* = 0.091), and *RUNX1::RUNX1T1* (100% vs 69.2%, *p* = 0.096). The CR rate after one cycle did not differ between the two treatment groups in patients with AML bearing *IDH1/2* (90.9% vs 70%, *p* = 0.293) or *FLT3‐TKD* mutations (87.5% vs 66.7%, *p* = 0.538). In addition, the CR rate after one cycle between two groups was 100% (2/2) vs 83.3% (5/6) (*p* = 1.000), 75% (3/4) versus 0% (0/1) (p = 0.400), 0% (0/1) vs 50% (1/2) (*p* = 1.000) in the DAV and the DA groups in patients with *CBFβ::MYH11*, *NUP98* rearrangement, or a *TP53* mutation, respectively—this is hard to compare with the limited patients in these oncogenic backgrounds (Table S4, Supporting Information).

To better understand the target population for DAV regimens and enhance therapy, we conducted comprehensive phosphoproteomics to characterize the kinase features in patients with AML who exhibited sensitivity to DAV regimens but were resistant to DA treatment. Additionally, we aimed to explore the mechanisms underlying DAV resistance and identify potential methods to overcome and further strengthen the efficiency of the DAV regimen.

Our analysis revealed that more phosphoproteins were associated with DAV resistance rather than with DA treatment (Figure 1E), suggesting the involvement of multiple biological processes in DAV resistance. We defined a kinase signature of DAV‐sensitive patients based on the presence of differential phosphoproteins between DA responders and non‐responders but did not show differential expression in DAV responders compared to non‐responders. Several biological processes, including chromatin organization, RNA processing, the cell cycle, and DNA/RNA metabolism, have been shown to contribute to DAV sensitivity. Notably, phosphoproteins enriched in the “chromatin organization” term showed relatively higher expression in both DA‐NR and DAV‐CR (Figure 2E,F), indicating its critical role in AML. Thus, chromatin regulation is a critical target in AML.^[^ ^35^ ^]^

Furthermore, our kinase‐substrate analysis identified AURKB, a serine/threonine kinase involved in mitosis regulation, which has been reported to be a potential inducer of DA resistance contributing to tumor progression and drug resistance in solid tumors while restoring sensitivity upon addition of venetoclax (DAV). Among the enriched kinases associated with DAV sensitivity, CDK2, an inhibitor that has been widely employed to combat drug resistance in AML,^[^ ^17^, ^36^, ^37^ ^]^ emerges as another potential predictor worthy of further discussion regarding its potential contribution to DA resistance, which may be overcome by DAV treatment.

We found that ectopically expressed AURKB triggered resistance to DA treatment, whereas DAV treatment restored sensitivity (Figure 3). Thus, our study revealed that AURKB activity represents a potential guideline for the application of the DAV regimen in patients with AML. Elevated AURKB expression is associated with adverse cytogenetic abnormalities and high white blood cell counts in patients with AML.^[^ ^38^ ^]^ Additionally, AURKB inhibition has been shown to effectively inhibit AML‐related cell proliferation.^[^ ^39^ ^]^ These studies provide substantial evidence for the pivotal role of AURKB in AML progression.

Additionally, we discovered that AKT1 is the predominant kinase contributing to DAV resistance in AML, and its inhibition effectively overcame the unsatisfactory anti‐leukemic activity and induced robust cell death when combined with DAV treatment. Our primary objective was to elucidate the distinctive phosphoproteomic signature of patients with DA‐NR who exhibited responsiveness to the DAV regimen, in contrast to the DA‐CR group. To achieve this, we conducted a comparative analysis of the three groups, excluding the DAV‐NR, and a comprehensive analysis centered on the AURKB. We then used phosphorylated H3 S10 as an indicator for validation, specifically within the DA‐CR/DA‐NR/DAV‐NR groups. Subsequently, we compared DAV‐NR and DAV‐CR to explore the mechanism of resistance unique to the DAV regimen, which may not be directly linked to AURKB but may involve other kinases such as AKT1.

During phosphorylated motif analysis, “xxxxxx_S_DxExxx” emerged as a highly enriched within the cohort of proteins associated with DAV resistance, which corresponds to the CK2 kinase consensus motif.^[^ ^40^ ^]^ This suggests that CK2 may contribute to DAV resistance. This observation was consistent with our subsequent analysis (Figure 4H,I).

Age among the four groups was statistically different in this study (Table 1), and further investigations were conducted to explore whether age could potentially influence DAV sensitivity or resistance. We examined the protein levels of AURKB and AKT1 in our AML proteome datasets (unpublished) and performed western blot analysis on samples from patients with AML (Figure S3I,J, Supporting Information). The analysis revealed no apparent correlation between AURKB or *AKT1* expression and patient age, suggesting that age is unlikely to be a contributing factor to DAV response variability among patients with AML.

Overall, our quantitative phosphoprotein datasets unraveled a novel kinase signature of populations, vulnerable to the DAV regimen, shedding light on the underlying mechanism of DAV resistance and providing a potential strategy to overcome and improve the efficacy of the DAV regimen. These findings will contribute to the advancement of precision medicine for AML treatment and pave the way for further research to optimize therapeutic strategies for patients with this challenging disease.

## Conflict of Interest

The authors declare no conflict of interest.

## Author Contributions

J. J. and S. H. contributed equally to this work. JJ designed the study, collected data, and revised the manuscript; SYH collected data, performed experiments, and analyzed results; YYY and MML collected data, and participated in result analysis; TZ conducted PCA analysis; LPM, MY, and HYT participated in data collection; JYH read and provided input on the manuscript; YHZ and HFW designed the study, conducted result analysis, wrote the manuscript, and provided administrative support. YHZ specifically performed proteomics data analysis. All the authors reviewed and approved the final version of the manuscript.

## Supporting information

Supporting Information

Supporting Information

Supporting Information

## Data Availability

The data that support the findings of this study are openly available in OMIX at https://ngdc.cncb.ac.cn/omix, reference number 4738.
