# Supplementary material for: Phosphoproteomic Characterization and Kinase Signature Predict Response to Venetoclax Plus 3+7 Chemotherapy in Acute Myeloid Leukemia
Source: Adv Sci (Weinh). 2023 Dec 31;11(11):2305885. doi: 10.1002/advs.202305885 (PMC10953567; doi:10.1002/advs.202305885)
Supplement: Supplementary file 1 — Supporting Information [file ADVS-11-2305885-s001.pdf]

ADVANCED SCIENCE
Open Access

Supporting Information

for *Adv. Sci.*, DOI 10.1002/advs.202305885

Phosphoproteomic Characterization and Kinase Signature Predict Response to Venetoclax Plus 3+7 Chemotherapy in Acute Myeloid Leukemia

Jie Jin, Shangyu Hou, Yiyi Yao, Miaomiao Liu, Liping Mao, Min Yang, Hongyan Tong, Tao Zeng, Jinyan Huang, Yinghui Zhu and Huafeng Wang**

Supplementary Materials

Table S1. Clinical characterization of complete remission (CR) and non-response (NR) AML cohorts treated with DA or DAV regimen used for phosphoproteomics analysis.

Patient No.	Gender	Age	WBC (10^9/L)	N (10^9/L)	HGB (g/L)	PLT (10^9/L)	BM Blasts (%)	FAB Subtype	Mutations*	Fusion Genes	Karyotype	Karyotype Risk Stratification	ELN Risk Stratification
DA-CR-1	F	32	28.5	1.4	91	13	67	M5	FLT3, CBL, STAG2	RUNX1::RUNX1T1	46,XX,t(8;21)(q22;q22),del(9)(q13;q31)[16]/46,XX[4]	favorable	favorable
DA-CR-2	F	25	7	0.7	103	58	53	M4	FLT3, KIT, NF1, NRAS	CBFβ::MYH11	46,XX[20]	intermediate	favorable
DA-CR-3	F	38	82.8	1.7	64	29	92	M1	KRAS, NRAS, PTPN11, WT1, GATA2	Negative	46,XX[20]	intermediate	adverse
DA-CR-4	M	28	48.92	15.7	134	95	88	M5	FLT3-ITD, NPM1, DNMT3A	Negative	46,XY[20]	intermediate	intermediate
DA-CR-5	M	42	17.93	2.15	91	18	72	M5	KIT, RUNX1	RUNX1::RUNX1T1	46,XY,t(8;21)(q22;q22)[10]/46,XY[10]	favorable	favorable
DA-CR-6	M	33	65.6	21	67	39	82	M4	FLT3-ITD	Negative	46,XY[20]	intermediate	intermediate
DA-CR-7	F	21	153.5	13.8	104	20	50	M5	Negative	KMT2A::MLLT3	46,XX[20]	intermediate	intermediate
DA-CR-8	M	41	58.21	0.88	73	34	72	M4	CEBPA	Negative	Undetectable	intermediate	favorable
DA-CR-9	M	38	2.83	0.08	66	87	92	M1	NPM1, IDH1	Negative	Undetectable	intermediate	favorable
DA-CR-10	F	36	0.5	0.85	58	18	87	M5	EP300, TIN, TTN, TET2	KMT2A::MLLT4	47,XX,der(21)(p11),+der(21)(p11)[12]/46,XX[8]	intermediate	adverse
DA-NR-1	M	33	23.76	5.01	95	91	82	M2	FLT3-ITD	Negative	46,XY[20]	intermediate	intermediate
DA-NR-2	M	58	110.89	10.31	91	11	82	M5	ASXL1	KMT2A::MLLT3	46,XX[20]	intermediate	intermediate
DA-NR-3	F	28	0.61	0.09	67	99	78	M5	IDH1	Negative	46,XY,-7[8],-9[10],-14[10],+1–3mar[14]/46,XY[6]	adverse	adverse
DA-NR-4	F	44	17.14	6.96	64	16	63.5	M5	ASXL1	RUNX1::RUNX1T1	45,X,-Y,t(8;21)(q22;q22)[12]/46,XY[8]	favorable	favorable
DA-NR-5	F	51	10.46	4.88	116	37	66	M5	KIT D816V	RUNX1::RUNX1T1	45,XY,t(8;21)(q22;q22)[12]/46,XY[8]	favorable	favorable
DA-NR-6	F	37	141.9	4.3	86	28	82	M2	Negative	Negative	46,XX[20]	intermediate	intermediate
DA-NR-7	F	57	41.71	0.5	72	17	66	M2	CEBPA, KRAS, NRAS, GATA2	Negative	46,XX[20]	intermediate	favorable
DAV-CR-1	M	30	4.2	0.3	75	113	81.5	M5	KMT2A-MLLT3	KMT2A::MLLT3	46,XY,t(9;11)(p22;q23)[8]/91,XXYY,-81[2]/46,XY[10]	intermediate	intermediate
DAV-CR-2	M	32	209.85	10.86	114	29	92	M5	TTN, EPPK1, MUC16	KMT2A::MLLT4	46,XY,del(11)(q23)[20]	adverse	adverse
DAV-CR-3	F	42	54.43	6.19	119	187	63	M5	NPM1, IDH2, DNMT3A, PTPN11, NRAS, EPPK1, TTN, ASDL1, CSF3R	Negative	46,XY[20]	intermediate	favorable
DAV-CR-4	M	19	50.6	0.5	109	18	82	M5	CEBPA	Negative	46,XY,del(9)(q21;q32)[8]/46,XY,del(1)(q11)[2]/46,XY[10]	intermediate	favorable
DAV-CR-5	F	48	105.4	26.03	118	85	82	M5	FLT3-ITD, WT1	Negative	46,XX[20]	intermediate	adverse
DAV-CR-6	M	20	35.66	1.89	73	24	91	M1	Negative	RUNX1::RUNX1T1	46,XY[20]	favorable	favorable
DAV-CR-7	F	22	130.2	95.03	104	87	66	M5	Negative	NUP98::HOXA13	47,XX,i(7q),der(11)(p25),+12[20]	adverse	adverse
DAV-CR-8	M	49	60.73	1.82	123	37	70	M5	CBFβ, KIT	Negative	46,XY[20]	intermediate	favorable
DAV-CR-9	F	47	32.3	1.9	84	24	90	M1	KDM6A	Negative	46–48,XX,-1,der(3)(p26),+4[5],-7[3],-11,+3mar[6]/46,XX[4]	adverse	adverse
DAV-CR-10	F	35	19.37	0.19	88	13	70	M2	CEBPA, WT1	Negative	46,XX[20]	intermediate	favorable
DAV-NR-1	M	46	40.54	19.26	77	36	46.5	M5	KRAS	Negative	46,XY[20]	intermediate	adverse
DAV-NR-2	M	32	31.43	10.37	61	74	66.5	M5	FLT3, IDH2, NPM1, DNMT3A, FLT3	Negative	46,XY[20]	intermediate	intermediate
DAV-NR-3	M	53	67.75	0.27	82	16	89	M5	FLT3-TKD, STAG2, ARID1B, CEBPA, WT1, PTPRT, TTN, NCOR2, TET2	Negative	46,XY[20]	intermediate	intermediate
DAV-NR-4	F	58	30.32	1.2	85	105	62	M0	ASXL1, IDH1, BCORL1, TET2	Negative	46,XX,t(4;12)(p11;p13)[18]/46,XX[2]	intermediate	adverse
DAV-NR-5	M	64	11.07	4.04	70	115	67	M2	FLT3-ITD, IDH2, NF1	NUP98::NSD1	46,XY[20]	intermediate	intermediate
DAV-NR-6	M	50	42.04	23.81	145	19	27	M4	CBL, GATA2, RUNX1	Negative	46,XY[20]	intermediate	adverse

DA, Darubicin+Cytarabine; DAV, Darubicin+Cytarabine+Venetoclax; WBC, white blood cell count; N, neutrophil count; HGB, hemoglobin; PLT, platelet; BM, bone marrow; ELN Risk Stratification, The European LeukemiaNet Risk Stratification; F, female; M, male; Undetectable, no mitotic figures was observed; *, mutations were identified utilizing next generation sequencing integrated over the entire exon or hotspot regions of genes that are frequently mutated in myeloid malignancies.

Table S4. Therapeutic efficacy of DA and DAV treatments in patients with AML bearing diverse oncogenic backgrounds.

	DAV Group (n=72)	DA Group (n=78)	p value
FLT3-ITD	19	13	
CR	17 (89.5)	8 (61.5)	0.091
CRi	0	1 (7.7)	0.406
Composite CR	17 (89.5)	9 (69.2)	0.194
PR	0	0	NA
NR	2 (10.5)	4 (30.8)	0.194
ED	0	0	NA
NPM1	18	11	
CR	17 (94.4)	6 (54.5)	0.018
CRi	0	0	NA
Composite CR	17 (94.4)	6 (54.5)	0.018
PR	0	0	NA
NR	1 (5.6)	5 (45.5)	0.018
ED	0	0	NA
IDH1/2	22	10	
CR	20 (90.9)	7 (70)	0.293
CRi	0	1 (10)	0.312
Composite CR	20 (90.9)	8 (80)	0.572
PR	0	0	NA
NR	2 (9.1)	2 (9.1)	0.572
ED	0	0	NA
FLT3-TKD	8	6	
CR	7 (87.5)	4 (66.7)	0.538
CRi	0	0	NA
Composite CR	7 (87.5)	4 (66.7)	0.538
PR	0	0	NA
NR	1 (12.5)	2 (33.3)	0.538
ED	0	0	NA
RUNX1:RUNX1T1	13	13	
CR	13 (100)	9 (69.2)	0.096
CRi	0	1 (7.7)	1.000
Composite CR	13 (100)	10(76.9)	0.220
PR	0	1 (7.7)	1.000
NR	0	2 (15.4)	0.480
ED	0	0	NA
KMT2A rearrangement	6	6	
CR	6 (100)	2 (33.3)	0.061
CRi	0	1(16.7)	1.000
Composite CR	6	3 (50)	0.182
PR	0	0	NA
NR	0	2 (33.3)	0.455
ED	0	1 (16.7)	1.000
CBFβ-MYH11	2	6	
CR	2 (100)	5 (83.3)	1.000
CRi	0	0	NA
Composite CR	2 (100)	5 (83.3)	1.000
PR	0	0	NA
NR	0	1 (16.7)	1.000
ED	0	0	NA
NUP98 rearrangement	4	1	
CR	3 (75)	0	0.400
CRi	0	0	NA
Composite CR	3 (75)	0	0.400
PR	0	0	NA
NR	1 (25)	1 (100)	0.400
ED	0	0	NA
TP53	1	2	
CR	0	1 (50)	1.000
CRi	0	0	NA
Composite CR	0	1	1.000
PR	0	0	NA
NR	1 (100)	1 (100)	1.000
ED	0	0	NA

Numbers in the second and third column denote the number of patients, whereas numbers in parentheses denote percentages.

Figure S1

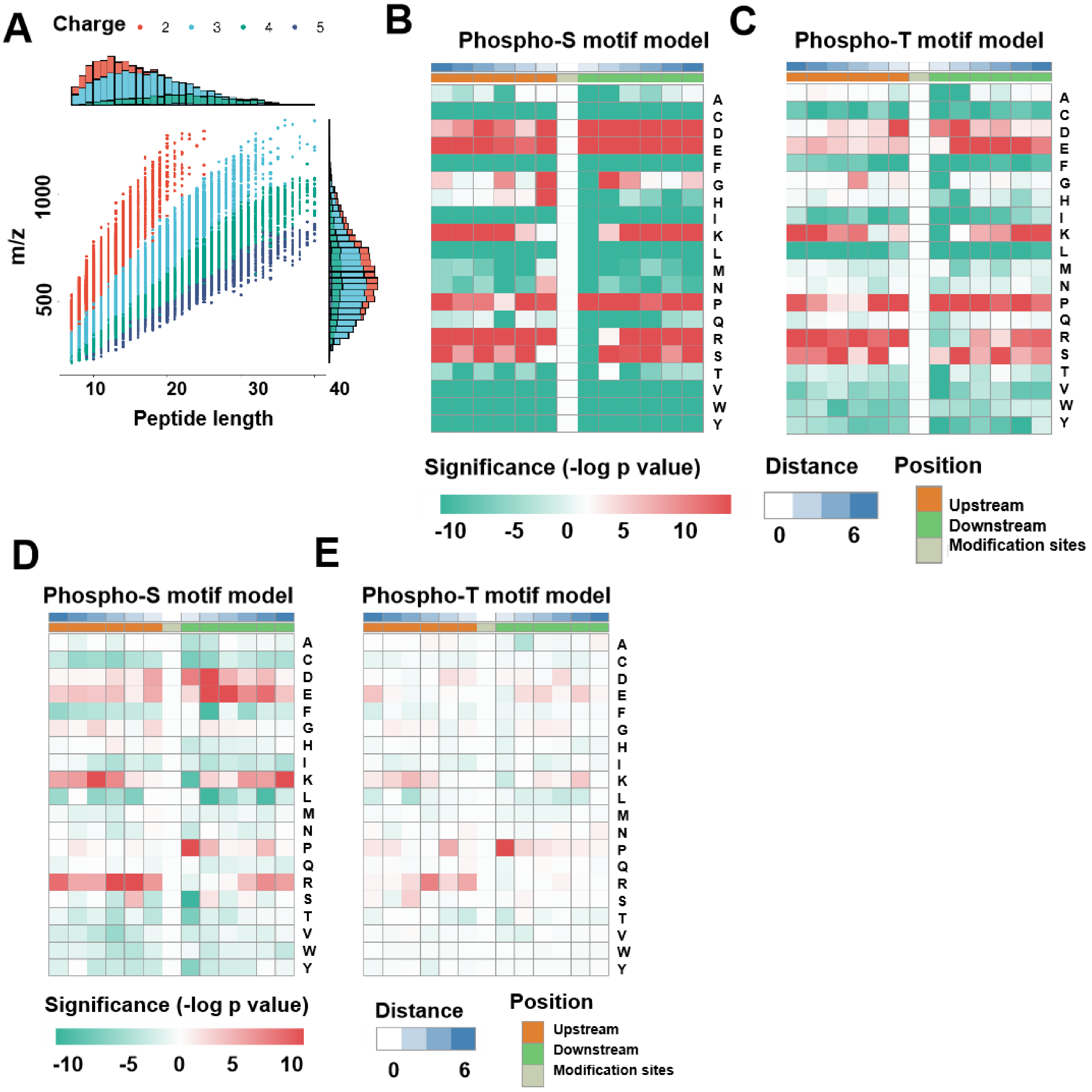

Figure S1. Quantitative phosphoproteomics profiling reveals a conserved signature linked to DAV regimen response in AML. (A) Length and charge of identified phosphopeptides in our datasets. (B–E) Phosphorylation motif enrichment heatmap of serine and threonine in total identified phosphorylated proteins (B and C) and differential phosphoproteins in DAV-NR/CR (D and E). The relative abundance of amino acid residues flanking the phosphorylation sites are represented by an intensity map. The intensity map shows the relative abundance for six amino acids starting from the phosphorylation site. The colors in the intensity map represent the $\log_{10}$ of the ratio of frequencies (red shows enrichment; green shows depletion).

Figure S2

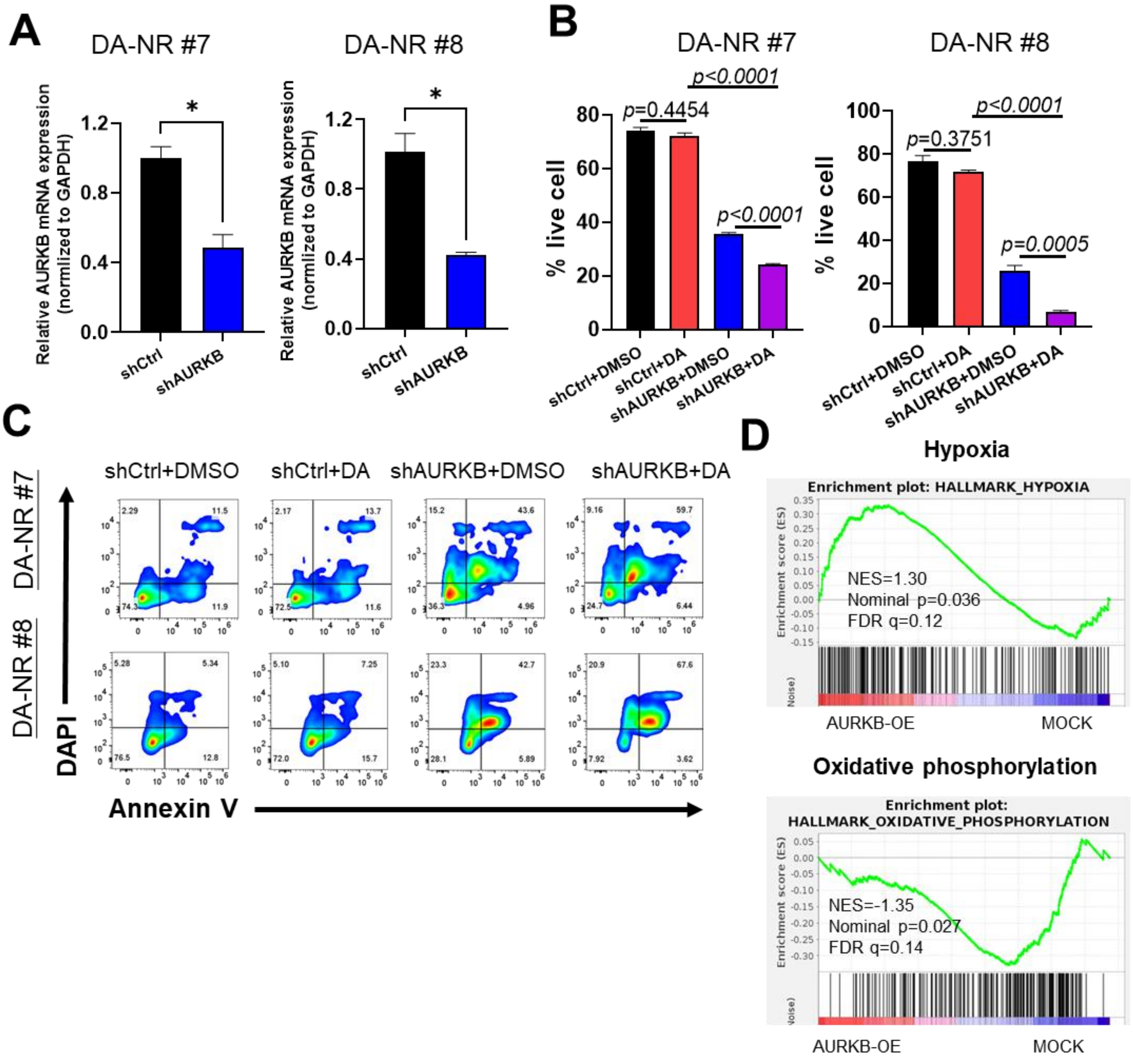

Figure S2. AURKB activation induces hypoxia upregulation and decreases oxidative phosphorylation. (A) Bar chart showing relative mRNA expression of AURKB (normalized to GAPDH) in shCtrl, or shAURKB transduced AML primary patients (DA-NR); (B) Bar chart showing the percentage of live cells indicated by Annexin V-APC/DAPI in shCtrl or shAURKB transduced AML primary patient cells, treated with DMSO or DA (D: 5nM; A:100 nM). (C) Representative flow cytometry profile of live or dead cells indicated by Annexin V-APC/DAPI in AML primary patient cells transduced with shCtrl or shAURKB and treated with DMSO or DA (D: 5 nM; A:100 nM). (D) Gene set enrichment analysis (GSEA) plots revealing that hypoxia was upregulated and oxidative phosphorylation was downregulated upon *AURKB* overexpression in THP1 cells.

Figure S3

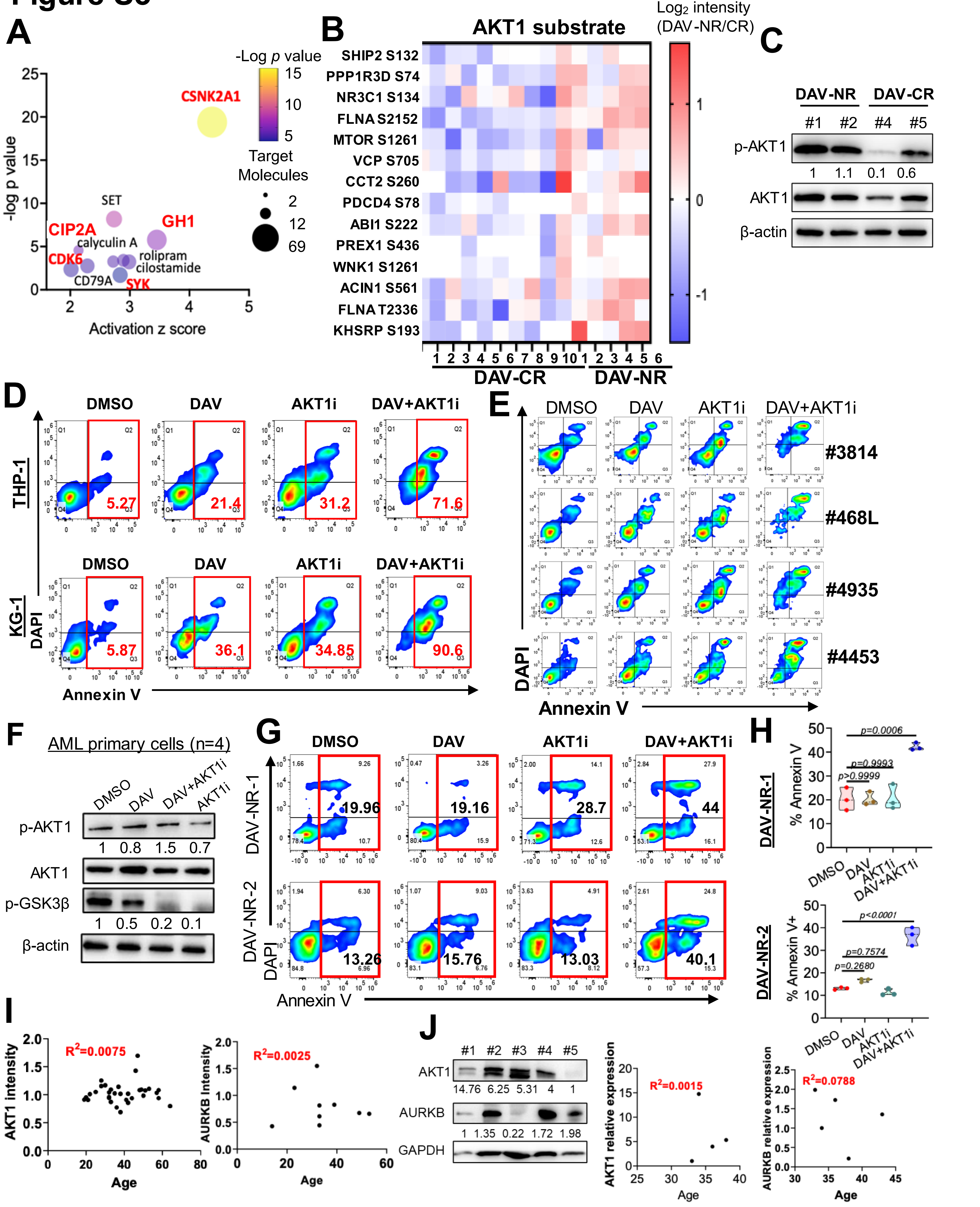

Figure S3. AKT1 activation is a specific marker of AML resistance to DAV treatment. (A) Predicted top 10 enriched kinases upon IPA analysis of differentially phosphorylated proteins. (B) Heatmap presents the normalized abundance of AKT1 substrates in differential phosphoproteins datasets. (C) Western blot analysis of p-AKT, AKT, and β-actin in DAV-CR and DAV-NR primary samples from patients with AML (AKT intensity was normalized based on *β-actin* expression; p-AKT intensity was normalized based on *AKT* expression). (D and E) Apoptosis assay of cell lines (n=2) and AML primary cells (n=4) after 48 h of AKT1 inhibitor and/or DAV treatment. (F) Immunoblot analysis of lysates from AML primary cells (n=4), showing the levels of phosphorylated AKT1 and GSK-3β proteins in the presence of DAV alone, AKT1 inhibitor alone, or DAV and AKT1 inhibitor for 48 h. Total AKT1 and β-actin were used as control. (G and H) Apoptosis assay of DAV-NR primary cells (n=2) after 48 h of AKT1

inhibitor and/or DAV treatment. (I) Correlation analysis between age and *AUKRB* or *AKT1* expression, based on two AML proteomics dataset (unpublished). (J) Immunoblot analysis of lysates from AML primary cells (n=5), showing the levels of AKT1 and AURKB. Protein intensity was quantified and correlated with the age of patients with AML.
